# The New Laboratories at the London School of Tropical Medicine

**Published:** 1911-08-19

**Authors:** 


### THE NEW LABORATORIES AT THE LONDON SCHOOL OF TROPICAL MEDICINE.

The new laboratories of the London School of Tropical Medicine, which are now fast approaching completion, present some unique features and are in every respect constructed and equipped to meet the latest requirements of medical science. The buildings adjoin the school within the grounds of the Branch Hospital of the Seamen's Hospital Society at Connaught Road, Albert Dock. These laboratories are designed exclusively for the departments of helminthology and protozoology. A separate floor is set aside for each, and every detail is arranged to afford facilities for the study and teaching of these subjects. The buildings, which consist of two floors and a flat roof, are constructed of ferro-concrete on the Hennibique system, from designs by Messrs. Young and Hall, Southampton Street, Bloomsbury. Each floor is divided into four rooms—a private laboratory, about eighteen by sixteen, with a supplementary sink-room, for the head of the department, a general laboratory for the accommodation of eight advanced students, which measures about twenty by twenty, and an attendant's room. These various apartments are equipped with broad teak benches, into which are let at frequent intervals small porcelain sinks. The private laboratory will also serve as the library for the special department, while the general laboratory is fitted as a museum for the particular subject taught. In the attendant's room are specially constructed incubators, and the entire flooring is of terrano. The flat roof is fitted with animal houses, cages, and other requirements of the laboratories below. Each floor, and the flat roof, is approached from the school grounds by an external iron staircase. The buildings present a very ordinary appearance, as they are lined with red brick to harmonise with the main structure of the school and the adjoining hospital, but, as a matter of fact, the construction is very different. The framework is of iron, and the floors, which are not more than 5 inches in thickness, are made of cement and thin iron rods. The main walls are 11 inches thick, which, together with the internal plaster, gives a total thickness of just over 1 foot, but in the centre of the walls there is a space of 2 inches, which insures a uniform temperature both in winter and summer. Louvre windows afford ventilation, while central fireplaces provide for ample heating for the entire wing. All water mains, gas pipes and electric light wires are carried on the surface of the walls.

These new additions have been necessitated by the demand for advanced instruction in helminthology and protozoology; an increasing number of students entering for the various courses each year. The students are principally English graduates, but there are a large number of foreign practitioners who are attracted to the school from every tropical colony and, indeed, from almost every part of the world. It is believed that these additions will provide ample accommodation for some time to come. The laboratories in the school used hitherto for these subjects in future will be used as a lecture theatre in connection with the new work.

About 140 students join the London School of Tropical Medicine every year, and many of them, after taking out the ordinary class, proceed to the advanced courses in one or other of the special departments. Under certain conditions veterinary graduates are admitted to the advanced courses, there being a working arrangement between the Royal Veterinary College and the school.

